# A Glance at the Development and Patent Literature of Tecovirimat: The First-in-Class Therapy for Emerging Monkeypox Outbreak

**DOI:** 10.3390/v14091870

**Published:** 2022-08-25

**Authors:** Mazen Almehmadi, Mamdouh Allahyani, Ahad Amer Alsaiari, Mohammed Kanan Alshammari, Abrar Saleh Alharbi, Khansa Hamza Hussain, Lojain Ibrahim Alsubaihi, Mehnaz Kamal, Shahad Saleh Alotaibi, Atheer Nasser Alotaibi, Afeefah Awaid Aldhafeeri, Mohd Imran

**Affiliations:** 1 Department of Clinical Laboratory Sciences, College of Applied Medical Sciences, Taif University, P.O. Box 11099, Taif 21944, Saudi Arabia; 2 Department of Pharmaceutical Care, Rafha Central Hospital, North Zone, Rafha 76312, Saudi Arabia; 3 Department of Pharmaceutical Sciences, Primary Healthcare Center, Mecca 24341, Saudi Arabia; 4 Department of Cardiac Sciences, College of Medicine, King Saud University, Riyadh 11472, Saudi Arabia; 5 Department of Pharmaceutical Care, Prince Sultan Armed Forces Hospital, Medina 42313, Saudi Arabia; 6 Department of Pharmaceutical Chemistry, College of Pharmacy, Prince Sattam Bin Abdulaziz University, Al-Kharj 11942, Saudi Arabia; 7 College of Pharmacy, Shaqra University, Dawadmi, Riyadh 17649, Saudi Arabia; 8 College of Pharmacy, Shaqra University, Shaqra 11961, Saudi Arabia; 9 Department of Nursing, North Area Armed Forced Hospital, King Khalid Military City, Hafr Al-Batin 31991, Saudi Arabia; 10 Department of Pharmaceutical Chemistry, Faculty of Pharmacy, Northern Border University, Rafha 91911, Saudi Arabia

**Keywords:** Tecovirimat, SIGA-246, TPOXX, orthopoxvirus, monkeypox, patents

## Abstract

Monkeypox disease (MPX) is currently considered a global threat after COVID-19. European Medicines Agency (EMA) approved Tecovirimat in capsule dosage form (200 mg) as the first treatment for MPX in January 2022. This article highlights Tecovirimat’s development and patent literature review and is believed to benefit the scientists working on developing MPX treatments. The literature for Tecovirimat was gathered from the website of SIGA Technologies (developer of Tecovirimat), regulatory agencies (EMA, United States Food and Drug Administration (USFDA), and Health Canada), PubMed, and freely accessible clinical/patent databases. Tecovirimat was first recognized as an anti-orthopoxvirus molecule in 2002 and developed by SIGA Technologies. The USFDA and Health Canada have also recently approved Tecovirimat to treat smallpox in 2018 and 2021, respectively. The efficacy of Tecovirimat was verified in infected non-human primates (monkeys) and rabbits under the USFDA’s Animal Rule. Most clinical studies have been done on Tecovirimat’s safety and pharmacokinetic parameters. The patent literature has revealed inventions related to the capsule, injection, suspension, crystalline forms, amorphous form, and drug combinations (Tecovirimat + cidofovir) and process for preparing Tecovirimat. The authors foresee the off-label use of Tecovirimat in the USA and Canada for MPX and other orthopoxvirus infections. The authors also trust that there is immense scope for developing new Tecovirimat-based treatments (new drug combinations with other antivirals) for orthopoxvirus and other viral diseases. Drug interaction studies and drug resistance studies on Tecovirimat are also recommended. Tecovirimat is believed to handle the current MPX outbreak and is a new hope of biosecurity against smallpox or orthopoxvirus-related bioterrorism attack.

## 1. Introduction

Monkeypox disease (MPX) is currently considered a global threat [1,2,3,4,5,6]. The zoonotic monkeypox virus (MPXV) (Family: Poxviridae; Subfamily: Chordopoxvirinae; Genus: Orthopoxvirus), a double-stranded DNA virus, causes MPX. The MPXV is related to the smallpox variola virus, also an orthopoxvirus [1,3,4,5,6]. The MPXV was first identified and isolated from monkeys in 1958 in Denmark [3,5,6]. Therefore, it is called MPXV. The first confirmed human MPX case was reported in 1970 in the Democratic Republic of Congo [3,4,5,6]. MPX is typical and endemic in African countries, including Cameroon, the Central African Republic, the Democratic Republic of Congo, Liberia, Nigeria, Sierra Leone, and Ghana. The first case of MPX outside Africa was reported in 2003 in the United States [3,4,5,6]. African rodents are thought to be the natural reservoir of MPXV, and MPXV infections have been reported in humans, monkeys, mice, rats, squirrels, and prairie [2,5,6]. In the past, outbreaks of MPX have occurred in the Democratic Republic of Congo (1997 and 2020), African regions (1997), the Central African Republic (2016), Nigeria (2017 and 2018), Cameroon (2018), Singapore (2019), the United Kingdom and Northern Ireland (2021), and the United States of America (2003 and 2021) [7]. In 2021–2022, the outbreak of MPX (about 1285 confirmed cases) had re-emerged in about 28 non-African countries comprising America (Argentina, Canada, Mexico, and the United States of America), Eastern Mediterranean (Morocco and the United Arab Emirates), Europe (Austria, Belgium, Czechia, Denmark, Finland, France, Germany, Hungary, Ireland, Israel, Italy, Latvia, Malta, Netherlands, Norway, Portugal, Slovenia, Spain, Sweden, Switzerland, and the United Kingdom), and Western Pacific (Australia). This outbreak is also present in African countries, with 59 confirmed cases of MPX and more than 1536 suspected cases [8].

The MPXV can be transmitted to humans by direct contact (oral, nasal, intradermal, etc.) with the body fluid, respiratory droplets, or skin lesion of the infected animal. Contact with contaminated fomites can also cause human MPXV infection. Human-to-human transmission is also possible. Therefore, household members and sexual partners are at a greater risk of developing MPX [6]. Two clades of MPXV (Central African or Congo Basin clade and West African clade) have been reported, wherein the Congo Basin clade is more virulent and transmissible than the West African clade [4,6,9]. It is thought that the reported cases of MPX in non-African countries are due to the West African clade of MPXV [4,9]. The MPXV replicates/multiplies at the infection site, causes local inflammation, primary viremia, and spreads to lymph nodes/lymphoid organs, leading to secondary viremia and symptoms of MPX [10]. The symptoms of monkeypox appear within 5–21 days. The symptoms of MPX include fever, chills, headache, muscle ache (myalgia), backache, exhaustion (asthenia), swollen lymph nodes (lymphadenopathy), and rashes (face, mouth, chest, hand, feet, anus, genitals, conjunctivae, etc.) [11,12,13]. MPX diagnosis is challenging because of its similarity to smallpox and chickenpox. Diagnosis of MPX can be made by PCR testing and sequencing analysis. However, the lymphadenopathy at the prodromal stage of MPX is considered an essential feature of MPX to distinguish MPX from smallpox and chickenpox [11,12,13]. If untreated, MPX can cause secondary infections, sepsis, bronchopneumonia, encephalitis, and corneal disease leading to loss of vision and death (3–6% cases) [12]. Some factors that are promoting the rapid MPXV spread across the globe include cessation of smallpox vaccination, increased interaction between humans and animals, increased travel to affected countries, decreased international coordination, difficulty in the diagnosis of MPX, lack of research on monkeypox, and poor availability of MPX treatments (supportive care, vaccine, cidofovir, and Tecovirimat) [14].

In January 2022, the European Medicines Agency (EMA) approved Tecovirimat as the first oral treatment for monkeypox. This article highlights Tecovirimat’s pharmaceutical development, clinical trials, inventions, and patent literature in a single document. The literature for Tecovirimat was gathered from the website of SIGA Technologies (developer of Tecovirimat), regulatory agencies (EMA, United States Food and Drug Administration (USFDA), and Health Canada), PubMed, and freely accessible clinical/patent databases. Tecovirimat, TPOXX, SIGA-246, SIGA246, ST-246, ST246, and SIGA Technologies, alone or in combination with other keywords (smallpox/monkeypox), were used to collect the literature from the sources mentioned earlier.

## 2. Tecovirimat

### 2.1. Chemistry

**Figure 1:**
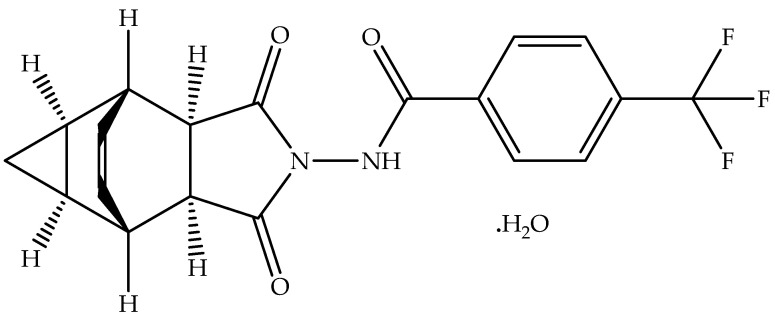
Chemical structure of Tecovirimat monohydrate.

Tecovirimat (Synonyms: TPOXX, SIGA-246, SIGA246, ST-246, ST246; CAS registry numbers: 816458-31-8, 869572-92-9; Molecular Formula: C_19_H_15_F_3_N_2_O_3_; Molecular Weight: 376.332; Chemical name: N-[(3aR,4R,4aR,5aS,6S,6aS)-3,3a,4,4a,5,5a,6,6a-octahydro-1,3-dioxo-4,6 ethenocycloprop[f]isoindol-2(1H)-yl]-4-(trifluoromethyl)benzamide) is a small tetracyclic acylhydrazide molecule used to treat orthopox viral infection, including monkeypox [15,16,17]. On 6 January 2022, the EMA approved the capsule dosage form (200 mg) of crystalline Tecovirimat monohydrate (Figure 1) (Molecular Formula: C_19_H_15_F_3_N_2_O_3_·H_2_O; Molecular Weight: 394.35; CAS registry number: 1162664-19-8) to treat monkeypox, smallpox, and cowpox, which are caused by viruses of the same family (orthopoxviruses) [16].

The chemical structure of Tecovirimat has some chiral carbons. Therefore, it exists in many stereoisomeric forms. However, only one stereoisomer is used in the approved dosage form of Tecovirimat (Figure 1) [18,19]. Tecovirimat monohydrate (a non-hygroscopic white crystalline solid) is insoluble in water at a pH range of 2.0–6.5 [19] but demonstrates high permeability in CaCo-2 cells. Accordingly, Tecovirimat monohydrate is classified as a BCS-II drug [18,19]. Tecovirimat exists in different polymorphic forms, including crystalline Tecovirimat monohydrate Form I, amorphous form, hemihydrate Form V, and other crystalline forms. The crystalline Tecovirimat monohydrate Form I is used to develop the marketed dosage form (capsule). Furthermore, the crystalline Tecovirimat monohydrate Form I can be prepared consistently by recrystallization from a mixture of ethyl acetate and water [18].

### 2.2. Worldwide Approval

Tecovirimat monohydrate has been approved by the EMA [16], USFDA [17], and Health Canada [20,21] to treat smallpox (Table 1). However, only EMA has approved Tecovirimat to treat monkeypox [16].

### 2.3. Development

**Figure 2:**
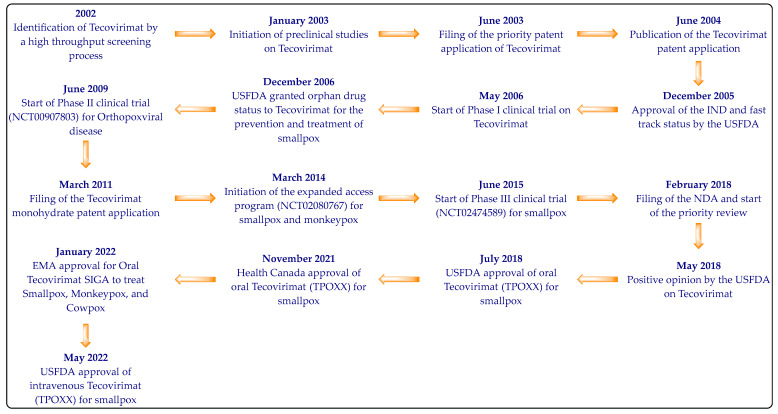
The development timeline of Tecovirimat.

Tecovirimat was recognized in 2002 as an effective molecule against orthopoxvirus-induced cytopathic effects by a high throughput screening process of 356,240 compounds [25,26]. The chemical structure of Tecovirimat has been provided first time in Patent Cooperation Treaty (PCT) Patent Application Publication Number WO2004112718A2 (previously owned by Viropharma) [27]. The crystalline Tecovirimat monohydrate used as the active ingredient of TPOXX was disclosed in 2011 for the first time in the United States Patent Application Publication number US2011236434A1, which has now been granted as US9339466B2 (SIGA Technologies) [28]. SIGA Technologies have developed TPOXX (capsule and intravenous dosage forms of Tecovirimat) in collaboration with the Biomedical Advances Research and Development Authority (BARDA) of the US Department of Health and Human Services. Tecovirimat was mainly developed to combat smallpox-related bioterror concerns [17,20,21]. SIGA Technologies is also developing Tecovirimat’s oral (liquid) formulations [17]. The development timeline of Tecovirimat is provided in Figure 2 [17,25].

### 2.4. Pharmacology

The in vitro antiviral activity-based concentration of Tecovirimat inhibiting the virus-induced cytopathic effect (CPE) by 50% (EC_50_ in μmol/L) was promising against different orthopoxvirus (Variola = 0.016–0.067; Monkeypox = 0.014–0.039; Rabbitpox = 0.015; Vaccinia = 0.009). It completely inhibited the CPE of the wild-type cowpox virus and extracellular vaccinia virus formation but had little effect on the intracellular formation of the vaccinia virus [17]. The clinical trials in humans with smallpox are neither feasible nor ethical. Therefore, the efficacy of Tecovirimat was verified in infected rabbits and non-human primates (monkeys) under the USFDA’s Animal Rule [17,29,30,31,32,33]. The effectiveness of Tecovirimat in the non-human primate monkeypox model utilizing MPX strain Zaire 79 (V79-I-005) infected cynomolgus monkeys is reported [29,30,31,32]. Tecovirimat (3, 10, 30, and 100 mg/Kg once a day) was administered orally to different groups of infected monkeys after three days of infection (stage of secondary viremia). The treatment duration was 14 days. Tecovirimat administration demonstrated 100% protection to the infected animal and reduced the viral load and lesion formation. The animal not receiving Tecovirimat died or required euthanasia after 33 days of the infection. An equivalent drug concentration level in animals and humans was observed at a dose of 10 mg/Kg/day and 400 mg/day, respectively. This observation suggested an adequate oral dose of 400 mg/day for 14 days to treat monkeypox or smallpox infections in humans [29,30,31,32]. The study of Tecovirimat in the rabbitpox virus model also demonstrated a 90–100% survival rate for rabbits when Tecovirimat (20 mg/Kg, 40 mg/Kg, 80 mg/Kg, and 120 mg/Kg) was administered for 14 days after starting the dose on day four after the infection [30,31,32].

**Figure 3:**
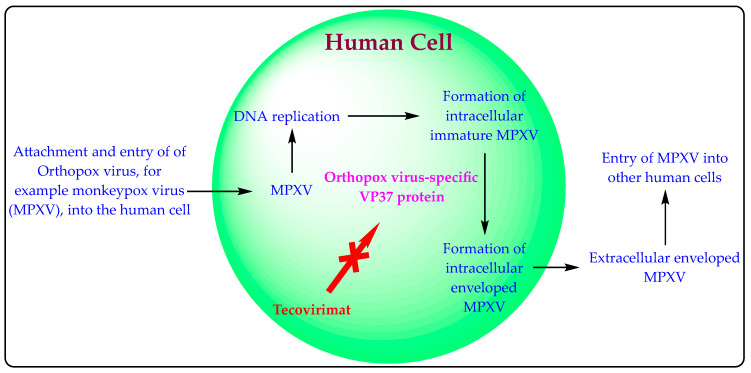
Mechanism of action of Tecovirimat.

The mechanism of action of Tecovirimat is well explained in the literature [17,25], documents released by SIGA Technologies [20,21], and the approved drug label of TPOXX [23,30,31]. Tecovirimat is an orthopoxvirus-specific VP37 protein inhibitor that inhibits the virus’s systemic spread to other cells [17,20,21,23,25,30,31]. VP37 protein was recognized as a target of Tecovirimat by the genetic mapping of Tecovirimat-resistant mutant viruses [25]. The orthopoxvirus-specific VP37 protein is responsible for forming an envelope around the orthopoxvirus-like MPXV. Creating this envelope is essential for exiting the virus from the cell and spreading the virus to other cells. The mechanism of action of Tecovirimat is also depicted in Figure 3.

The important pharmacological parameters of Tecovirimat are provided in Table 2.

## 3. Clinical Studies

The information about past and ongoing clinical studies on Tecovirimat was collected from the clinical database [36], employing different keywords. This search was done on 11 July 2022, and revealed many clinical studies on Tecovirimat (Tecovirimat = 11; SIGA-246 = 10; SIGA246 = 10; ST-246 = 11; ST246 = 11; TPOXX = 11). The overlapping results were removed, and the summary of the finalized 12 studies is provided in Table 3. The readers can open the page of the clinical database [36] and get the complete clinical study data using the National Clinical Trial number (NCT number) for each study mentioned in Table 3.

The clinical studies of Tecovirimat cited in Table 3 are related to smallpox (six studies), vaccinia (one study), healthy volunteers (one study), vaccinia (one study), orthopox viral disease (two studies), and monkeypox (two studies). No study has been conducted on children and pregnant women. In summary, the clinical studies of Tecovirimat were mainly related to its pharmacokinetic and safety parameters. Tecovirimat was well tolerated without any serious side effects in the phase I studies (NCT00728689 and NCT00431951). The phase II study (NCT00907803) revealed headache and nausea as Tecovirimat’s most frequent side effects without any serious effect on patients’ wellbeing. The phase III clinical study (NCT02474589) did not identify any safety concerns with the oral administration of Tecovirimat and demonstrated that Tecovirimat’s bioavailability increases up to 50% when taken with food. Our search on PubMed also revealed some pharmacokinetic studies on Tecovirimat [37,38,39,40,41]. The pharmacokinetic parameters of Tecoviramat are now well established and have already been discussed in Table 2. Accordingly, the authors have not elaborated on the studies related to pharmacokinetic parameters of Tecovirimate.

## 4. Patent Searching and Summary

Tecovirimat’s patents/patent applications were searched on 11 July 2022, through freely available patent databases (Espacenet, Patentscope, and the United States Patent and Trademark Office) [42,43,44,45,46]. A preliminary search employing different keywords and combinations thereof (Tecovirimat; TPOXX; SIGA246; ST-246 + Monkeypox; ST-246 + Smallpox; ST246 + Monkeypox; ST246 + Smallpox; SIGA-246 + Smallpox; SIGA-246 + Monkeypox; SIGA + Smallpox; SIGA + Monkeypox) provided hundreds of hits, which were not possible to discuss in this review. Tecovirimat has been developed by SIGA Technologies and is the marketing authorization holder for Tecovirimat in the USA, Europe, and Canada. Accordingly, the authors are providing a summary of patents/patent applications assigned to SIGA Technologies, including the patents listed in the Orange Book (OB) of the USFDA [47] and other important patents/patent applications explicitly claiming inventions of Tecovirimat (Table 4). It should be noted that most of the patent numbers mentioned in Table 4 belong to the same patent family. Therefore, they have identical authors and titles but different claims, patent numbers, and grant dates.

## 5. Discussion

MPX is the currently emerging global outbreak. The MPX is closely related but less fatal than smallpox disease [1,2,3,4,5,6]. Smallpox vaccination is thought to control MPX because the MPXV and smallpox virus are genetically close relatives and share common signs and symptoms. However, smallpox was eradicated long ago, and the routine administration of the smallpox vaccine has not been done by a large part of the global population [17]. This has increased the chances of individuals getting infected with MPX, making it a currently unmet medical need.

In January 2022, the EMA approved the Tecovirimat capsule (200 mg), developed by SIGA Technologies, as the first treatment for MPX [16]. Tecovirimat was mainly invented for treating smallpox because of bioterrorism concerns. The US Strategic National Stockpile has reserved about 2 million doses of Tecovirimat and other vaccines/drugs (JYNNEOS, ACAM2000, cidofovir, and vaccinia immunoglobulin intravenous (VIGIV)) in case of any bioterrorist attack [17]. The USFDA and Health Canada approved Tecovirimat for treating smallpox in 2018 and 2021, respectively, but not MPX [17,20,21]. Based on the EMA approval, the off-label use of Tecovirimat and the post-exposure prevention of MPX is foreseeable in the USA and Canada. These countries may also approve Tecovirimat for MPX shortly. The regulatory approval of Tecovirimat in other MPX-affected countries is also predictable soon. The EMA-, Health Canada-, and USFDA-approved capsules of Tecovirimat contain the crystalline Form I of Tecovirimat monohydrate because of its thermodynamic stability over other polymorphic forms [18,19]. Tecovirimat has been developed under the Animal Rule, which does not require efficacy-based clinical trials in humans for life-threatening, unnatural, and severe diseases. Accordingly, the efficacy of Tecovirimat was established in non-human primates (monkeys) and rabbits [17,29,30,31,32,33]. Tecovirimat also demonstrated high efficacy in different animal models against all recognized pathogenic orthopoxviruses at suggested human doses or its equivalents [81]. This is also evident from the clinical study data (Table 3), wherein most clinical studies were based on Tecovirimat’s pharmacokinetic and safety parameters. The drug–drug interaction, drug–disease interaction, and drug–food interaction studies are essential parameters concerning the safety and efficacy of a drug [82]. The interaction of Tecovirimat with repaglinide and midazolam has been established [30,31,32]. The contraindication of Tecovirimat injection in patients with renal injury is also reported [34]. Tecovirimat with milk, yogurt, applesauce, etc., is also recommended [30,31]. Tecovirimat has not shown any teratogenic effect in mice but showed non-teratogenic adverse events in maternal rabbits [18]. The authors believe that further studies must be done concerning drug interactions and teratogenic effects of Tecovirimat in higher animal models to ensure patient safety. Accordingly, the patient treated with Tecovirimat must be monitored, which may help identify its new benefits/side effect. Particular emphasis must be given while monitoring pregnant/pediatric/geriatric patients, patients suffering from chronic diseases such as diabetes, hypertension, cancer, etc., and patients on polypharmacy treatment. SIGA Technologies collaborates with many organizations to tackle the MPX outbreak [83]. Hopefully, this collaboration will also take care of the points mentioned earlier.

A DNA virus like MPXV has a lower mutation rate, but a mutation in the virus may develop resistance to a drug [2,81]. A nucleotide and frameshift mutation has been reported in the currently circulating strain of MPXV, which may increase its transmissibility [2]. Furthermore, mutations in MPXV are also possible. The authors opine to assess the efficacy of Tecovirimat against the MPXV that demonstrated nucleotide and frameshift mutation. This study may help to understand the possibility of Tecovirimat-resistance development by the MPXV. The case of Tecovirimat resistance among unresponsive patients to Tecovirimat must also be considered [3]. Tecovirimat is an inhibitor of the VP37 protein of orthopoxviruses [17,20,21,23,25,30,31]. The authors believe that drug repurposing-based and artificial intelligence-empowered drug development programs on the existing marketed antiviral drugs may also help to identify VP37 protein inhibitors for further development.

Developing commercially important inventions requires an understanding of existing patented inventions. SIGA Technologies owns many patents related to Tecovirimat (Table 4). These patents claim the marketed pharmaceutical compositions (capsule and intravenous injection) and suspension; method of treating orthopoxvirus infection; different polymorphic forms (crystalline and amorphous); and process for preparing Tecovirimat. SIGA Technologies is also developing an oral suspension formulation of Tecovirimat in collaboration with BARDA [17]. The synergistic drug combination patent application (Tecovirimat + cidofovir) has also been filed [78]. Tecovirimat does not interfere with the DNA replication process of MPXV and is unable to prevent the formation of intracellular MPXV (Figure 3). Accordingly, the authors trust that combining Tecovirimat with existing DNA replication inhibitors may provide synergistic effects. The effect of Tecovirimat may be reduced in immunocompromised patients (HIV/AIDS, leukaemia, lymphoma, generalized malignancies, etc.) [30,31,32]. Combining immunity boosters such as zinc, quercetin, vitamin C, thymoquinone, and other antioxidants [84,85,86] may increase Tecovirimat’s efficacy for MPX among immunocompromised patients. The authors believe that there is an excellent scope for developing more and better combinations of Tecovirimat against orthopoxvirus infections.

## 6. Conclusions

Tecovirimat is a new hope against the MPX outbreak and biosecurity against smallpox or orthopoxvirus-related bioterrorism attacks. At the time this article was written, Tecovirimat was only approved by the EMA to treat MPX. We expect the regulatory approval of Tecovirimat in other MPX-affected countries shortly. The efficacy of Tecovirimat in MPXV demonstrating nucleotide and frameshift mutation is warranted to assess the possibility of Tecovirimat resistance. Tecovirimat has been established as a safe and effective MPX treatment, but further Tecovirimat interaction-based pharmacological studies are also necessary to provide safe and effective treatment. Finally, the research programs on Tecovirimat-based drug combinations are also suggested to develop better MPX treatments.

## Figures and Tables

**Figure 4:**
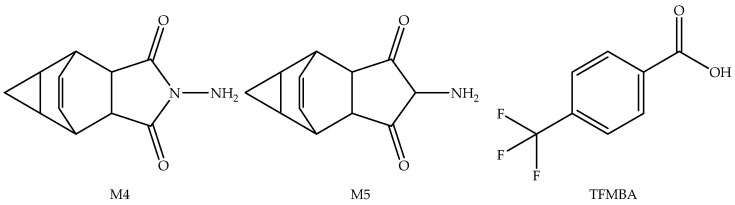
Pharmacologically inactive metabolites of Tecovirimat.

**Table 1:** R_x_ data of the EMA, USFDA, and Health Canada approved Tecovirimat.

Active Ingredient (Proprietary Name; Applicant)	Dosage Form (Route; Strength)	Approval Date (Marketing Status)	Exclusivity Code (Exclusivity Expiry Date)	Approved Indications
USFDA [22]				
Tecovirimat monohydrate (TPOXX; Siga Technologies Inc.)	Immediate-release capsule (Oral; 200 mg)	13 July 2018 (Prescription)	New Chemical Entity (13 July 2023); Orphan Drug Exclusivity for capsule (13 July 2025)	Human smallpox disease caused by the variola virus in adults and pediatric patients weighing at least 13 Kg (capsule)/3 Kg (intravenous)
	Solution (Intravenous; 10 mg/mL)	18 May 2022 (Prescription)		
EMA [16]				
Tecovirimat monohydrate (Tecovirimat SIGA; SIGA Technologies)	Immediate-release capsule (Oral; 200 mg)	6 January 2022 (Prescription)	Exclusivity information is not available. However, ten years of marketing exclusivity is possible	Smallpox, monkeypox, cowpox, and complications due to replication of vaccinia virus following vaccination against smallpox
Health Canada [23,24]				
Tecovirimat monohydrate (TPOXX; Siga Technologies Inc.)	Immediate-release capsule (Oral; 200 mg)	29 November 2021 (Prescription)	Marketing exclusivity expires on 29 November 2027	Smallpox disease in adults and pediatric patients weighing at least 13 Kg

**Table 2:** Important pharmacological parameters of Tecovirimat.

Parameter	Summary
Recommended dosage (Capsule 200 mg)	Adults: 600 mg two times a day; Pediatric patient (13 to <25 Kg): 200 mg two times a day; Pediatric patient (25 to <40 Kg): 400 mg two times a day; Pediatric patient (40 Kg or more): 600 mg two times a day; Treatment duration in all cases = 14 days; Capsule must be taken after half-hour of fatty meal which increases the absorption of the drug. The entire content present inside the capsule (recommended dose) can also be mixed carefully with milk (30 mL) or soft food (yogurt, applesauce, etc.) and administered to patients unable to swallow the capsule within 30 min [30,31].
Recommended dosage (Intravenous)	Pediatric patient (3 to <35 Kg): 6 mg/Kg body weight two times a day over 6 h; Other patients (35 to <120 Kg): 200 mg two times a day over 6 h; Treatment duration in all cases = 14 days [34].
Contraindications	None for capsule dosage form [30,31]. The intravenous injection should not be administered to individuals with serious renal impairments/injury [34].
Warning / Precautions	Tecovirimat should be used cautiously in individuals with compromised immunity/renal/liver functions. Tecovirimat may be less efficacious in immunocompromised individuals [30,31,32].
Adverse effects	Headache, dizziness, nausea, vomiting, diarrhea, and abdominal discomfort [30,31,32].
Established drug interactions	Co-administration of Tecovirimat with repaglinide/midazolam may lead to hypoglycemia/reduced effect of midazolam [30,31,32].
Absorption	T_max_ = 4–6 h [25,30,31]; The drug absorption increases by 39% with fatty meal [25,30,31].
Volume of distribution	After IV administration (200 mg) = 383 L; After oral administration (600 mg) = 1030 L; Blood to plasma ratio = 0.62–0.90; Protein binding = 77–82% [30,31,34].
Metabolism	Tecovirimat is a substrate of UGT1A4 and UGT1A1. Three important pharmacologically inactive metabolites, M4, M5, and TFMBA (Figure 4), have been identified. The glucuronide conjugates of Tecovirimat and M4 metabolite have been identified as the most abundant components in urine [31,34]. Tecovirimat and its M4 metabolites are inducers of CYP3A and CYP2B6. Therefore, the coadministration of Tecovirimat may reduce the therapeutic effect of drugs metabolized by CYP3A and CYP2B6 [31,34].
Elimination	Urine (mainly as Tecovirimat conjugate) = 73%; Faeces (mainly unchanged) = 23% [30,31,34].
Half-life	After IV administration (200 mg) = 21 h [34]; After oral administration (600 mg) = 19 h [30,31].
Clearance	After oral administration (600 mg) of Tecovirimat = 31 L/h; After intravenous administration (200 mg) of Tecovirimat = 13 L/h [30,34].
Overdose	LD_50_ (Single dose) = 2000 mg/Kg in mice/non-human primates [18,35]; No clinical overdose case reported [30,34]. The overdose cases must be monitored and handled based on signs and symptoms. Hemodialysis may not be an effective option for removing Tecovirimate from patients’ bodies [30,34].

**Table 3:** Summary of the clinical studies of Tecovirimat.

Study/Objective (Status; Condition; Intervention)	NCT Number (Sponsor & Collaborators; Location)	Clinical Phase (Study Type; Allocation; Numbers Enrolled; Comparison Group)	Primary Purpose (PP); Primary Outcome (PO); Study Starts Date (SSD); Study Completion Date (SCD); Last Update (LU); Results
Assessment of safety, tolerability, and pharmacokinetics of oral Tecovirimat (Completed; Orthopoxviral Disease; 400 mg and 600 mg Tecovirimat daily for 14 days)	NCT00907803 (SIGA Technologies and National Institutes of Health; United States)	2 (Interventional; Randomized; 107; Matching Placebo capsules)	PP: Safety and tolerability of Tecovirimate; PO: Change in the pre-determined safety parameters; SSD: June 2009; SCD: January 2010 LU: 21 September 2010; Results: Available
Protocol to treat orthopoxvirus infection (Available; Monkeypox and smallpox; 600 mg daily dose of Tecovirimat)	NCT02080767 (U.S. Army Medical Research and Development Command; United States)	Not mentioned (Expanded Access; Not mentioned; Not mentioned; Not mentioned)	PP: Expanded Access; PO: Not mentioned; SSD: Not mentioned; SCD: Not mentioned; LU: 11 February 2021; Results: Not available
Animal Regulatory Rule-based study to evaluate the safety, tolerability, and pharmacokinetics of Tecovirimat (Completed; Smallpox; 600 mg of Tecovirimat two times a day for 45 days)	NCT02474589 (SIGA Technologies and BARDA; United States)	3 (Interventional; Randomized; 449; Matching placebo capsule)	PP: Treatment; PO: Number of participants showing adverse effects; SSD: 19 June 2015; SCD: 24 August 2016; LU: 28 November 2017; Results: Available
Comparative safety and pharmacokinetic parameter evaluation of Form I and Form V of Tecovirimat (Completed; Monkeypox and smallpox; Administration of 400 mg of Form I and Form V of Tecovirimat for 13 days)	NCT00728689 (SIGA Technologies and National Institutes of Health; United States)	1 (Interventional; Randomized; 12; Groups administered with crystalline Form I (1–3 days) and Form V (4–13 days) and vice versa)	PP: Treatment; PO: Pharmacokinetic parameters of Form I vs. Form V; SSD: August 2008; SCD: October 2008; LU: 29 June 2015; Results: Available
Treatment of orthopoxvirus infection with intravenous Tecovirimat (Available; orthopoxvirus infection; Intravenous Tecovirimat (10 mg/mL))	NCT05380752 (U.S. Army Medical Research and Development Command and SIGA Technologies; United States)	Not mentioned (Expanded Access; Not mentioned; Not mentioned; Not mentioned)	PP: Treatment; PO: Not mentioned; SSD: Not mentioned; SCD: Not mentioned; LU: 19 May 2022; Results: Not available
Observational study on smallpox patients treated with Tecovirimat capsules (Enrolling by invitation; Smallpox; Tecovirimat (600 mg) two times a day for 14 days)	NCT03972111 (SIGA Technologies and BARDA; United States)	4 (Observational; Observational; 100; Not mentioned)	PP: Safety and survival status of patients; PO: Patient survival status after 44 days post the first dose; SSD: 1 January 2020; SCD: 30 September 2024; LU: 14 February 2022; Results: Not available
Evaluation of the safety, tolerability, and pharmacokinetics of Tecovirimat (Completed; Healthy volunteers; Single oral dose (250 mg, 400 mg, or 800 mg) for 21 days in fed healthy volunteers)	NCT00431951 (SIGA Technologies and National Institute of Allergy and Infectious Diseases; United States)	1 (Interventional; Randomized; 30; Matching placebo capsule)	PP: Treatment; PO: Changes in the pre-determined safety/tolerability parameters; SSD: February 2007; SCD: February 2008; LU: 27 July 2017; Results: Available
Animal Regulatory Rule-based evaluation of the safety, tolerability, and pharmacokinetics of Tecovirimat (Recruiting; Smallpox; Three capsules (3 × 200 mg) two times a day for 28 days)	NCT04971109 (SIGA Technologies and United States Department of Defense; United States)	3 (Interventional; Randomized; 445; Matching placebo capsule)	PP: Treatment; PO: Laboratory / physical test, Heart / respiratory parameters, C_max_/T_max_; SSD: 29 March 2022; SCD: 30 June 2023; LU: 27 May 2022; Results: Not available
Drug interaction study of oral Tecovirimat in Healthy subjects (Not yet recruiting; Smallpox; Single oral dose (600 mg) of Tecovirimat co-administered with a single oral dose of sevelamer carbonate (1600 mg tablet), sucroferric oxyhydroxide (500 mg tablet), calcium acetate (1334 mg tablet), or lanthanum carbonate (500 mg tablet))	NCT04485039 (SIGA Technologies, BARDA, and PPD; United States)	4 (Interventional; Randomized; 44; Not mentioned)	PP: Drug interaction study; PO: Plasma parameters of Tecovirimat; SSD: 15 May 2022; SCD: 23 April 2023; LU: 14 January 2022; Results: Not available
Safety and pharmacokinetic parameter evaluation of oral Tecovirimat when administered for 28 days (Recruiting; Smallpox; Oral Tecovirimat (600 mg) two times a day for 28 days and a single subcutaneous dose (0.5 mL) of JYNNEOS vaccine on day one and day 29)	NCT04957485 (SIGA Technologies and United States Department of Defense; United States)	2 (Interventional; Randomized; 445; Matching placebo of oral Tecovirimat and JYNNEOS vaccine)	PP: Treatment; PO: Geometric mean titer of vaccinia virus-neutralizing antibodies; SSD: 5 January 2022; SCD: 31 October 2022; LU: 27 May 2022; Results: Not available
Determination of the pharmacokinetic profile of Tecovirimat in individuals weighing > 120 Kg (Completed; Smallpox; Oral Tecovirimat capsule (200 × 3 = 600 mg) two times a day for seven days in subjects weighing > 120 Kg)	NCT04392739 (SIGA Technologies and BARDA; United States)	4 (Interventional; Not mentioned; 34; Not mentioned)	PP: Dosing regimen for people > 120 Kg; PO: Pharmacokinetic parameters after days 1, 2, 6, 8, and 9; SSD: 19 July 2019; SCD: 5 December 2019; LU: 21 May 2020; Results: Available
Tecovirimat to treat smallpox (Withdrawn; Vaccinia; Protocol not provided)	NCT00303225 (National Institute of Allergy and Infectious Diseases and National Institutes of Health Clinical Center; United States)	1 (Interventional; Not mentioned; Zero; Not mentioned)	PP: Treatment; PO: Not mentioned; SSD: 13 March 2006; SCD: 4 August 2006; LU: 2 July 2017; Results: Not available

**Table 4:** Summary of the critical patents/patent applications related to Tecovirimat.

S. No.	Patent/Application Number (Status)	Summary of the Claims
**Patents/** **Patent Applications Filed by SIGA Technologies**		
1	US9339466B2 (Patented case)	This OB-listed patent claims a crystalline polymorphic Form I of Tecovirimat monohydrate, its pharmaceutical composition, and a method of preparing polymorphic Form I of Tecovirimat monohydrate [28].
2	US8124643B2 (Patented case)	This OB-listed patent discloses Tecovirimat and claims a pharmaceutical composition containing a therapeutically effective amount of Tecovirimat and a pharmaceutically acceptable carrier [48].
3	US7737168B2 (Patented case)	This OB listed patent claims a method of preventing/treating orthopoxvirus infection (monkeypox, smallpox, cowpox, vaccinia, buffalopox, camelpox, elephantpox, and rabbitpox) employing a therapeutically effective amount (0.125–250 mg/Kg/day) of Tecovirimat [49].
4	US8802714B2 (Patented case)	This OB-listed patent claims a method of preventing/treating orthopoxvirus infection utilizing an adequate amount of a mixture of Tecovirimat (95% *w*/*w* or more) and one of its isomers (5% or less *w*/*w*) [50].
5	US8530509B2 (Patented case)	This OB-listed patent claims a pharmaceutical composition comprising an adequate amount of a mixture of Tecovirimat (95% *w*/*w* or more) and one of its isomers (5% or less *w*/*w*) for preventing/treating orthopoxvirus infection [51].
6	US10576165B2 (Patented case)	This OB-listed patent claims a unit liquid formulation containing Tecovirimat (2 to 20 mg/mL) and hydroxypropyl-β-cyclodextrin (125 to 400 mg/mL) [52].
7	US9233097B2 (Patented case)	This OB-listed patent claims a liquid formulation comprising an adequate amount of Tecovirimat, hydroxypropyl-β-cyclodextrin, and sufobutyl-ether-β-cyclodextrin [53].
8	US9907859B2 (Patented case)	This OB-listed patent claims to treat orthopoxvirus infection or eczema utilizing a liquid composition (injectable or topical) comprising Tecovirimat and hydroxypropyl-β-cyclodextrin, and sulfobutyl-ether-β-cyclodextrin [54].
9	US8039504B2 (Patented case)	This OB-listed patent claims a pharmaceutical composition (capsule) containing Tecovirimat and additional ingredients. It also claims a unit pharmaceutical composition containing Tecovirimat (200 mg), lactose monohydrate, croscarmellose sodium, colloidal silicon dioxide, hydroxypropyl methylcellulose, sodium lauryl sulfate, magnesium stearate, and microcrystalline cellulose [55].
10	US7956197B2 (Patented case)	A process for preparing Tecovirimat [56].
11	US9744154B2 (Patented case)	A polymorphic Form II of Tecovirimat and its preparation method [57].
12	US10045964B2 (Patented case)	A polymorphic Form III of Tecovirimat and a method to prepare it [58].
13	US10406137B2 (Patented case)	A polymorphic Form IV of Tecovirimat [59].
14	US10933050B2 (Patented case)	A polymorphic Form VI of Tecovirimat and a method of its preparation [60].
15	US9045418B2 (Patented case)	A process for preparing Tecovirimat [61].
16	US10124071B2 (Patented case)	A process for making a liquid formulation comprising Tecovirimat (including polymorphic Form I, Form II, Form III, Form IV, Form V, and Form VI) and cyclodextrin [62].
17	US10864282B2 (Patented case)	A process for preparing water-soluble lyophilized formulation of Tecovirimat with cyclodextrin [63].
18	US10716759B2 (Patented case)	A method of reducing the particle size of dehydrated Tecovirimat monohydrate (Form I) [64].
19	US10406103B2 (Patented case)	A method of hydrating Tecovirimat monohydrate (Form I) [65].
20	US9670158B2 (Patented case)	A method of preparing amorphous Tecovirimat [66].
21	US9889119B2 (Patented case)	A method of preparing amorphous solid dispersion of Tecovirimat by the spray-drying process [67].
22	US10155723B2 (Patented case)	A method of producing Tecovirimat [68].
23	US10662155B2 (Patented case)	A method of producing Tecovirimat [69].
24	US9546137B2 (Patented case)	A method of producing Tecovirimat [70].
25	US9862683B2 (Patented case)	A method of producing Tecovirimat [71].
26	US7687641B2 (Patented case)	A method of producing Tecovirimat [72].
27	AU2012268859B2 (Patented case)	A method of producing Tecovirimat [73].
28	US2021212987A1 (Notice of Allowance mailed)	A dry suspension of polymorphic Form I of Tecovirimat and simethicone and its use to treat orthopoxvirus infection [74].
**Important Patents/Patent Applications Filed by Other Applicants**		
29	US11369587B2 (Patented case)	This patent is assigned to the Institute of Pharmacology and Toxicology Academy of Military Medical Sciences, China. It claims an injectable composition comprising Tecovirimat, cyclodextrin, and meglumine [75].
30	US11318115B2 (Patented case)	This patent is assigned to the Institute of Pharmacology and Toxicology Academy of Military Medical Sciences, China. It claims a composition comprising Tecovirimat, cyclodextrin, and meglumine [76].
31	CN102406617B (Patented case)	This patent is assigned to the Institute of Bioengineering, The Academy of Military Medical Sciences, China. It claims a suspension composition comprising Tecovirimat, lactose, sodium carboxymethyl cellulose, dimethyl silicone oil, silica gel, and aspartame [77].
32	US8642577B2 (Patented case)	This patent is filed by Almond et al. It claims a method of treating pox virus infection using cidofovir in combination with Tecovirimat [78].
33	EP2202297B1 (Patented case)	This patent is assigned to Genelux Corporation. It claims to use Tecovirimat to treat an adverse side effect associated with cancer oncolytic pox virus therapy [79].
34	CN101445478B (Patented case)	This patent is assigned to the Institute of Biological Engineering, Academy of Military Medical Sciences, China, and claims a process for making Tecovirimat monohydrate [80].

## Data Availability

Not applicable.
